# PMP–diketopiperazine adducts form at the active site of a PLP dependent enzyme involved in formycin biosynthesis†
†Electronic supplementary information (ESI) available. See DOI: 10.1039/c9cc06975e


**DOI:** 10.1039/c9cc06975e

**Published:** 2019-11-15

**Authors:** Sisi Gao, Huanting Liu, Valérie de Crécy-Lagard, Wen Zhu, Nigel G. J. Richards, James H. Naismith

**Affiliations:** a Research Complex at Harwell , Didcot , OX11 0FA , UK; b BSRC , University of St Andrews , St Andrews , KY16 9ST , UK; c Department of Microbiology , University of Florida , Gainesville , FL 32611 , USA; d Department of Chemistry and California , Institute for Quantitative Biosciences , University of California , Berkeley , CA 94720 , USA; e School of Chemistry , Cardiff University , Park Place , Cardiff , CF10 3AT , UK; f Foundation for Applied Molecular Evolution , Alachua , FL 32415 , USA; g Division of Structural Biology , University of Oxford , Oxford , OX3 7BN , UK . Email: naismith@strubi.ox.ac.uk; h The Rosalind Franklin Institute , Didcot , OX11 0FA , UK; i State Key Laboratory of Biotherapy , University of Sichuan , China

## Abstract

ForI is a PLP-dependent enzyme from the biosynthetic pathway of the C-nucleoside antibiotic formycin. Cycloserine is thought to inhibit PLP-dependent enzymes by irreversibly forming a PMP–isoxazole. We now report that ForI forms novel PMP–diketopiperazine derivatives following incubation with both d and l cycloserine. This unexpected result suggests chemical diversity in the chemistry of cycloserine inhibition.

## Abstract 2

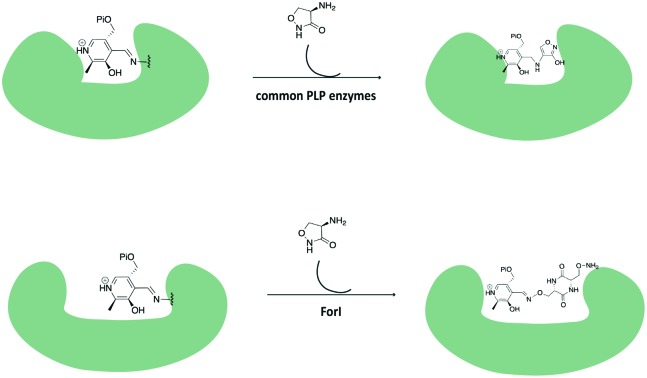
ForI is a PLP-dependent enzyme from the biosynthetic pathway of the C-nucleoside antibiotic formycin.

## 

As part of investigations into the C-nucleoside formycin A (8-aza-9-deazaadenosine, Fig. 1) biosynthetic gene cluster from *Streptomyces kaniharaensis*,^1^ we have characterised a pyridoxal 5′-phosphate (PLP) dependent aminotransferase (ForI) that aminates a pathway intermediate. C-Nucleosides are very interesting potential medicinal compounds that in addition to formycin A^2^–^4^ include showdomycin,^5^ minimycin^6^ and pyrazomycin.^7^ Schramm and colleagues, inspired by C-nucleosides, devised immunocillins^8^ which have considerable promise in treating a variety of serious diseases.^8^–^10^ C-Nucleosides are stable to acid and phosphorolysis by purine nucleoside phosphorylase,^11^ and thus may retain the utility of N-nucleosides known to have important medicinal activities while having improved pharmacokinetic properties.^12^–^14^

**Fig. 1:**
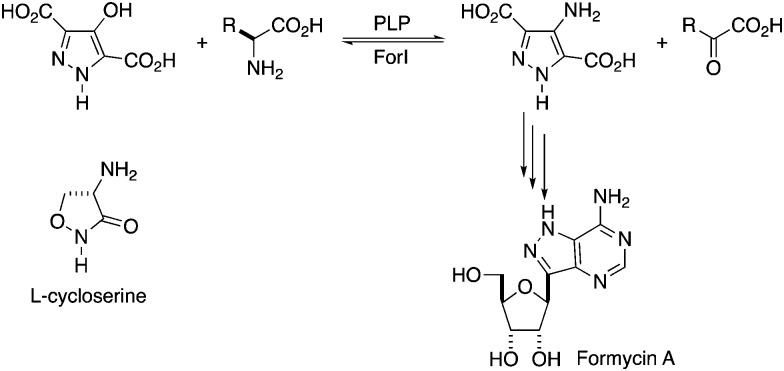
Proposed reaction catalysed by ForI. R = CH_2_COO^–^ for l-Asp or (CH_2_)_2_COO^–^ for l-Glu.^21^l-Cycloserine is shown.

Given that the heterocyclic substrate of ForI remains unconfirmed and not available, we probed the behaviour of ForI with d- and l-cycloserine, which are well known inhibitors of PLP-dependent enzymes, including transaminases,^15^ racemases^16^ and decarboxylases.^17^d-Cycloserine is a natural product used as a broad spectrum antibiotic,^18^ whose primary target is the bacterial PLP-dependent alanine racemase.^19^,^20^ Both enantiomers of cycloserine are known to form external aldimine pyridoxamine 5′-phosphate (PMP) adducts, which convert irreversibly to stable PMP–isoxazoles. We now report that cycloserine forms an adduct with ForI but unexpectedly it is a novel PMP–diketopiperazine adduct.

Recombinant ForI was overexpressed in *Escherichia coli* and purified by metal affinity and gel filtration chromatography.^22^ The enzyme purifies with bound PLP and exists as a dimer (Fig. S1 and Table S1, ESI†). Crystals were obtained in the presence of PLP and the structure solved to 1.18 Å resolution (Fig. 2a) by molecular replacement using the thermostable omega-transaminase from *Sphaerobacter thermophilus* (PDB: ; 5D95) as a model (Table S2, ESI†).

**Fig. 2:**
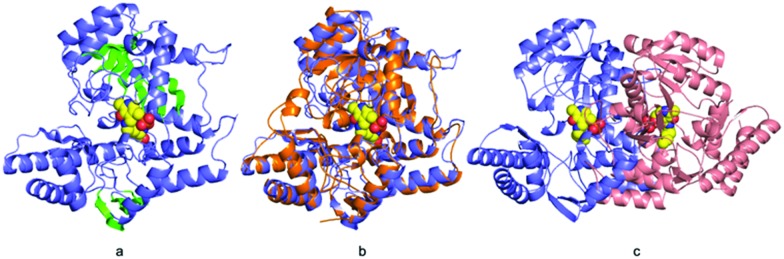
Structure of ForI. (a) Cartoon representation monomer of ForI, the conserved insertions characteristic of aspartate aminotransferases are highlighted in green. PLP is shown in spheres, carbon yellow, nitrogen blue and oxygen red. (b) ForI colored as (a), superimposed on glutamate-1-semialdehyde aminomutase in orange. (c) ForI is a dimer, the other monomer is colored salmon, PLP is colored as (a).

Residues 3 to 417 were located experimentally (Fig. 2a); the missing nine residues we assume to be conformationally flexible. ForI belongs to the aspartate aminotransferase (A-AT) superfamily with 50% sequence similarity to PLP-dependent glutamate-1-semialdehyde aminotransferase (PDB: ; 3K28) from *Bacillus anthracis* (Fig. S2, ESI†).^23^ Structurally^24^,^25^ ForI most closely resembles glutamate-1-semialdehyde aminomutase (GSAAT) (PDB: ; 2GSA).^26^ ForI possesses the mixed β-sheet and three-stranded antiparallel β-sheet insertion characteristic of the PLP dependent aspartic aminotransferase family (Fig. 2a).^27^ GSAAT and ForI share 30% sequence identity, and pairwise superposition gives an r.m.s.d. of 1.3 Å over 422 Cα atoms (Fig. 2b). Electron density shows that PLP forms a Schiff base (internal aldimine) with the active site Lys 262, (Fig. 3a).^28^ The dimer (Fig. 2c) arises from crystal symmetry.^29^,^30^ The predicted active site of ForI comprises residues from two monomers (Fig. 3b), indicating the dimer to be the functional unit. The resolution of the structure allows for visualisation of extensive hydration at the active site and water–PLP hydrogen bonds. PLP makes multiple contacts with the protein, broadly similar to those described for the GSAAT structure.^31^ In GSAAT residues Ala 161 to Ser 173 form an α-helix whose N-terminus that partly form the active site. The equivalent residues in ForI adopt a different arrangement creating a cavity (pocket) adjacent to PLP. The substitution of Met 248 in GSAAT by Gly 238 in ForI also leads to a more open active site (Fig. S3, ESI†). The catalytically important Glu 406 in GSAAT^26^ has no functional equivalent in ForI. The equivalent region, centred on Ser 389 in ForI, adopts a different conformation. In ForI, Trp 206 and Arg 207 which are not present in GSAAT, only partially fill the pocket. The consequence of these changes is that the pocket adjacent to the co-factor is larger in ForI (Fig. 3c).

**Fig. 3:**
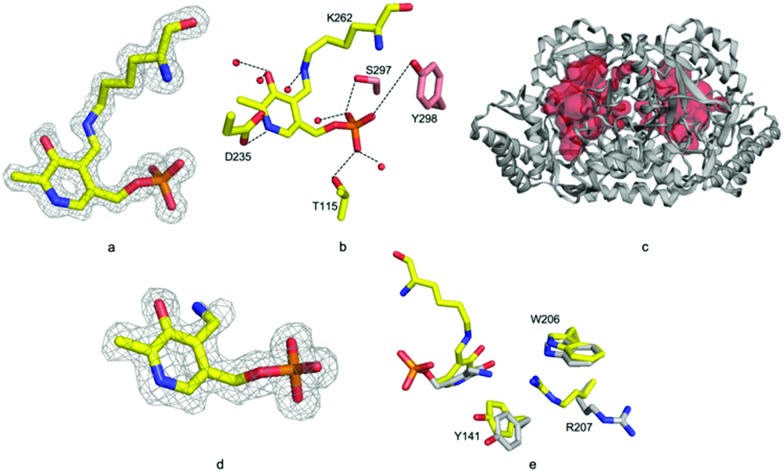
ForI active site (a) unbiased Fo–Fc map contoured at 3*σ* for the internal aldimine. (b) Hydrogen bonding interaction of PLP with surrounding water molecules and other residues. Residues from monomer A have carbons in yellow; monomer B salmon. (c) The ForI cavity is shown in red. (d) Unbiased Fo–Fc map contoured at 3*σ* for PMP. (e) PMP is rotated relative to PLP form resulting in conformational changes in active site residues. ForI–PLP carbons in yellow; ForI–PMP grey.

Incubating ForI with l-Glu shows a rapid change in the UV-vis spectrum (Fig. S4, ESI†). We incubated crystals of ForI with l-Glu for 20 minutes and collected a 1.56 Å resolution data set. The electron density clearly indicates that the pyridoxal co-factor is unattached to the enzyme, suggesting it to be PMP (Fig. 3d). Despite numerous attempts we have not observed density for l-Glu or α-KG. PMP forms a 3.2 Å hydrogen bonding interaction with ε-N of Lys 262. The plane of the PMP molecule has rotated by 22° relative to PLP (Fig. 3e). Residues Tyr 141 and Trp 206 are slightly displaced, Arg 207 switches to an alternate conformation (Fig. 3e). Accompanying these changes in protein structure are shifts in water molecule locations. These structural changes result in the pocket adjacent to the cofactor (bounded by Ala 25, Thr 60, Tyr 141, Trp 206) enlarging further and becoming more open to solvent.

Incubation of ForI with either d- or l-cycloserine immediately shows changes in the UV-vis spectrum (Fig. 4a and b). Spectral changes for the l-enantiomer appear to complete faster (<1 h) than the d enantiomer (<30 h). After incubating the protein at 4 °C with either 10 mM d or l cycloserine overnight, the protein was crystallised at pH 8 with 10 mM cycloserine, crystals were harvested on day 3 and 1.5 Å diffraction data collected (Table S2, ESI†). Mass spectrometry (Fig. S8, ESI†) and unbiased electron density (Fig. 4c and d) identified the formation of external aldimine PLP–diketopiperazine adducts, that have retained the stereochemistry of the cycloserine enantiomer used in soaking.

**Fig. 4:**
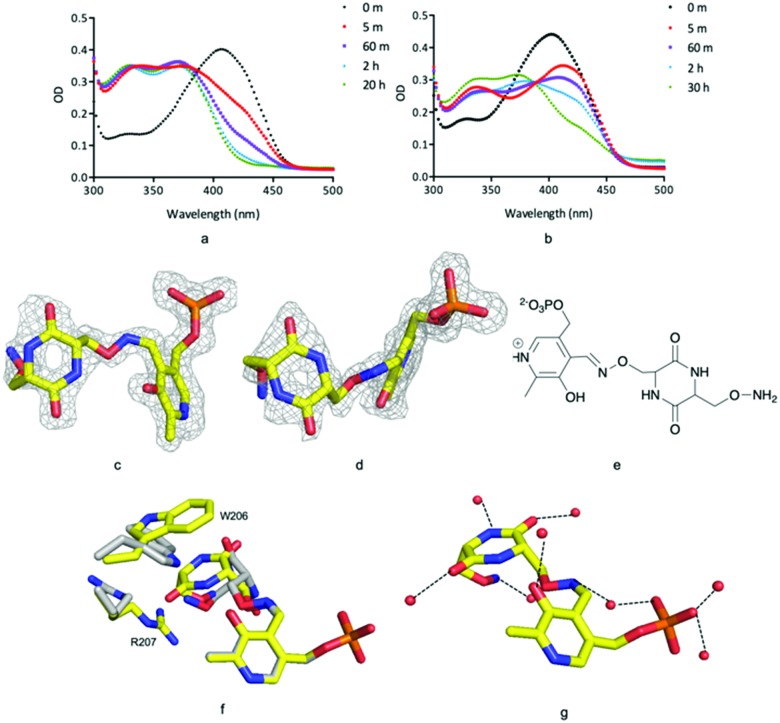
ForI and cycloserine. (a) UV-vis spectrum with l-cycloserine (b) UV-vis spectrum with d-cycloserine (c) unbiased Fo–Fc map contoured at 3*σ* PMP–diketopiperazine from l-cycloserine. (d) Unbiased Fo–Fc map contoured at 3*σ* for PMP–diketopiperazine from d-cycloserine. (e) Chemical structure of PMP–diketopiperazine adduct. (f) Active site amino residues exhibit different configuration for l-configured (yellow carbons) and d-configured (grey carbons) diketopiperazine rings. (g) l-Configured diketopiperazine adduct hydrogen bonds with water molecules as red spheres.

In each external aldimine complex, the atoms of the diketopiperazine adduct originating from PLP adopt the same positions as observed for the PMP structure. Tyr 141 adopts the same conformer seen in the PMP structure. In both structures the diketopiperazine ring occupies the pocket adjacent to the cofactor (Fig. 4f and g). However, the orientation and thus the details of the interactions with the protein of the diketopiperazine rings in this pocket are different (Fig. 4g).

In the l-cycloserine derived complex Trp 206 has adjusted its conformation in order to make π-stacking interactions with the diketopiperazine ring (Fig. 4f). Arg 207 adopts a different conformation than seen in either PLP or PMP structures, in this conformation it makes only a few van der Waals contacts with the adduct. The polar atoms in diketopiperazine ring make a series of hydrogen bonds to water molecules which bridge to the protein (Fig. 4g). The diketopiperazine ring arising from d-cycloserine does not π-stack with Trp 206 which is found in the same conformation seen in the PMP complex. The residue does make van der Waals interactions with the diketopiperazine but many fewer than seen in the l-configured adduct. Arg 207 adopts the conformer seen in the PMP structure and has no contact with the adduct (Fig. 4f). Tyr 141 makes more interactions with the d-configured diketopiperazine ring than were seen for Try 141 with the diketopiperazine ring that arose from l-cycloserine. The polar atoms of this adduct also make hydrogen bonds to water molecules (Fig. S5, ESI†). The diketopiperazine molecule may thus partly mimic the external aldimine of the aromatic planar heterocyclic ForI substrate (Fig. 1) and the possibility of π-stack with Trp 206 is especially suggestive.^32^ We note that the diketopiperazine binding pocket does not exist in GSAAT where Ser 163, Asn 217 and Glu 406 would clash with the molecule (Fig. S5, ESI†) and may reflect the difference in substrate.

The structure of GSAAT with PMP and gabaculine (PDB: 3GSB) has been reported.^26^ Gabaculine is located in a pocket adjacent to PLP but this location in ForI is blocked by Arg 23 (Fig. S5, ESI†). The more distantly related *Streptomyces fradiae* enzyme NeoB (PDB: ; 6CBL) (24% sequence identity, 1.94 Å r.m.s.d. 337 Cα's) (Fig. S2, ESI†), catalyses the amination of aminoglycoside antibiotics and the external aldimine with the d-neosamine has been reported.^33^ The sugar ring attached to the cofactor sits in the same pocket as the diketopiperazine but makes different interactions. The other sugar would clash with the main chain at Asn 152 in ForI (Fig. S6, ESI†) and both diketopiperazines would clash with Tyr 341 of NeoB.

The observation of PMP–diketopiperazine rather than PMP–isoxazole adduct was unexpected. The formation of the PMP–isoxazole proceeds *via* an external aldimine followed by tautomerisation which is catalysed by the key lysine (Scheme 1).^34^ Two related questions thus arise; why has a diketopiperazine not been observed before and why do we observe the PMP–diketopiperazine adduct?

**Scheme 1:**
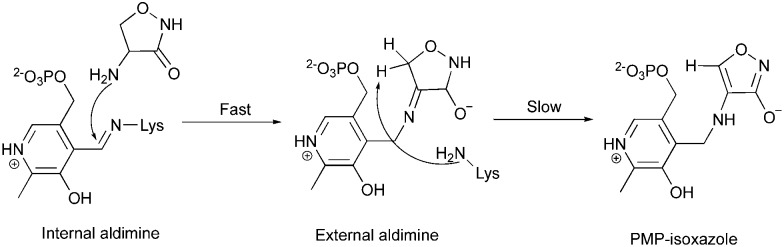
The formation of the PMP–isoxazole from d-cycloserine (adopted from ref. 34).

The structure of the PMP–isoxazole adduct has most recently been reported for the PLP-dependent *Mt*IlvE enzyme (PDB: ; 5U3F) from *Mycobacterium tuberculosis* which catalyses the l-glutamate-dependent transamination of α-ketoisocaproate, α-keto-methyloxopentanoic acid and α-ketoisovalerate.^34^*Mt*IlvE has a very different structure to ForI (10% sequence identity, 4.8 Å r.m.s.d. 71 Cα's) (Fig. S2, ESI†). Using only the atoms in the pyridoxal rings to superimpose the structures shows that the PLP environment is very different in the two enzymes and a diketopiperazine adduct would clash with residues in *Mt*IlvE. Other PLP d-cycloserine adduct complexes (; 2RJH, ; 1I2L, ; 1D7S, ; 5FAJ, ; 4IUT, ; 3TCM, ; 4D9F) have been reported with the non-aromatic d-configured external aldimine whilst others (; 3E6E, ; 1VFS, ; 1EPV, ; 2DAA) contain what appears, in the light of *Mt*IlvE study^34^ to be the PMP–isoxazole. Complexes following l-cycloserine incubation (; 4OMA, ; 1D7U, ; 1VFT; 1NIU, ; 1MDZ) show the PMP–isoxazole although one (; 4D9E) includes a non-aromatic, l-ketamine. Desulfhydrase racemase, lyase, and alanine aminotransferase enzymes (; 4D9E, ; 4D9F, ; 1VFT, ; 1VFS, ; 3E6E, ; 2RJH, ; 1EPV, ; 1I2L, ; 2DAA, ; 5FAJ, ; 4LUT, ; 3TCM) have very different proteins structures, thus as with *Mt*IlvE the atoms of pyridoxal rings rather than those in protein were used to superimpose the structures. Of these structures, only the active sites of ; 4D9E, ; 4D9F, ; 2RJH, ; 1VFS, ; 1VFT, ; 1I2L, ; 2DAA and ; 4LUT seem large enough (or sufficiently exposed to solvent) to accommodate a diketopiperazine adduct but would require some structural adjustments of side chains. Where the fold is sufficiently conserved to permit protein superposition (; 1D7S, ; 1D7U, ; 4OMA, ; 1NIU, ; 1MDZ), differences in the loops at the active site give rise to clashes that would require large main chain movements to accommodate a PMP–diketopiperazine. These structural difference may explain why diketopiperazine adducts were not reported for these enzymes.

Why is a PMP–diketopiperazine adduct seen in ForI? The pH, times and concentrations of protein and cycloserine used in our crystallisation and mass spectrometry experiments are comparable to other studies.^34^ Neither mass spectrometry nor NMR analysis of fresh l-cycloserine solution shows diketopiperazine; the supplier reports less than 1% impurity. Diketopiperazine forms spontaneously in cycloserine solutions; at 25 °C, pH 6 and 10 mM concentration the half-life of cycloserine is 10 h.^35^ The estimated half-life of cycloserine under crystallisation conditions (pH 8 and 4 °C) and mass spectrometry conditions (pH 8 and 4 °C) is >1000 h.^35^ The crystallisation experiment used 10 mM cycloserine with 1 mM protein for 84 h and mass spectrometry 2 mM cycloserine in solution with 0.1 mM protein for 14 h. The oxime of diketopiperazine is expected to be more reactive than the amine of cycloserine towards PLP. It is plausible that the diketopiperazine adducts arise from diketopiperazine present in solution that preferentially react with PLP.

The alternative explanation is that conversion of cycloserine to diketopiperazine is catalysed by ForI. PLP has been reported to catalyse diketopiperazine formation.^36^ A plausible chemical mechanism is proposed in Scheme 2. The initial step is formation of the external aldimine with cycloserine, common to all PLP enzymes. In ForI, we suggest this aldimine is stable enough and the active site large enough for a second molecule to enter the active site and ring open the cycloserine external aldimine (Scheme 2) before the irreversible PMP–isoxazole forms. We note that the positively charged Lys 262 is positioned such that it could stabilise the negatively charged tetrahedral intermediate that ring opening steps create (Scheme 2) and thus catalyse this reaction. The ring opened aldimine would then undergo a series of re-arrangements facilitated by proton shuttling and charge stabilisation. The large number of water molecules and hydrophilic residues at the active site, would be expected to assist this process. The difference in the rates of formation of the adducts, we suggest would arise from the favourable π-stacking interaction with Trp 206 seen only for l-configured diketopiperazine. Irrespective whether diketopiperazine is formed in solution or catalyzed by ForI, we conclude it is the enzyme's large polar cavity that favors formation the PMP–diketopiperazine adduct.

**Scheme 2:**
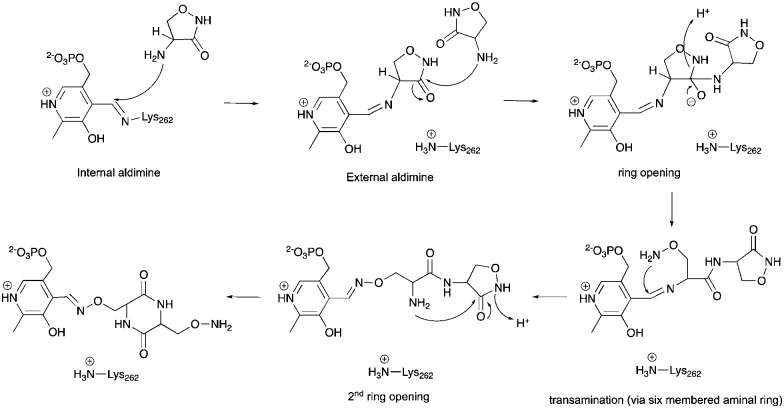
A hypothetical chemical mechanism for ForI-catalysed formation of the PMP–diketopiperazine adduct from l-cycloserine.

The covalent inhibition of PLP enzymes by cycloserine is at the heart of an antimicrobial agent. Prevailing wisdom is that this inhibition results from formation of a PMP–isoxazole adduct. A study of the SPT family of PLP enzymes proposed the SPT enzyme catalysed ring opening of the external cycloserine aldimine by water.^37^ Interestingly, diketopiperazine has been reported to inhibit PLP dependent aspartate–glutamate transaminase^38^ and to be active against *M. tuberculosis*.^39^ We suggest that PLP/cycloserine chemistry remains far from a fully understood process and it is likely there will be more surprises.

We thank Mr Thomas Williams for mass spectrometry, acquired using LCMS equipment funded by the EPSRC (EP/L027240/1). JHN is funded by the ERC (TNT-NCB 339367), NGJR by the BBSRC (BB/P018017/1) and VdCL by NIH (R01GM129793). JHN is a visiting scholar at Sichuan University.

## Conflicts of interest

There are no conflicts to declare.

## Supplementary Material

Supplementary information
