# Supplementary material for: PMP–diketopiperazine adducts form at the active site of a PLP dependent enzyme involved in formycin biosynthesis
Source: Chem Commun (Camb). 2019 Nov 15;55(96):14502–5. doi: 10.1039/c9cc06975e (PMC6927412; doi:10.1039/c9cc06975e)
Supplement: Supplementary file 1 [file CC-055-C9CC06975E-s001.pdf]

Electronic Supplementary Material (ESI) for ChemComm.
This journal is © The Royal Society of Chemistry 2019

Electronic Supplementary Information for

PMP-diketopiperazine adducts form at the active site of a PLP dependent enzyme involved in formycin biosynthesis.

Sisi Gao[a,b,c], Huanting Liu[c], Valerie de Crecy-Lagard[d], Wen Zhu[e], Nigel GJ Richards[f,g], James H Naismith[h,i*]

a. School of Chemistry, University of St Andrews, St Andrews, KY16 9ST, UK
b. Research Complex at Harwell, Didcot, OX11 0FA, UK.
c. Biomedical Sciences Research Complex, University of St Andrews, St Andrews, KY16 9ST, UK
d. Department of Microbiology, University of Florida, Gainesville, FL 32611, USA.
e. Department of Chemistry and California Institute for Quantitative Biosciences, University of California, Berkeley, CA 94720, USA
f. School of Chemistry, Cardiff University, Park Place, Cardiff, CF10 3AT, UK
g. Foundation for Applied Molecular Evolution, Alachua, FL 32415, USA
h. Division of Structural Biology, University of Oxford, Oxford, OX3 7BN, UK
i. The Rosalind Franklin Institute, Didcot, OX11 0FA, UK

Table of Contents

Supplementary figures 2
Supplementary tables 6
Method and materials 8
References 11

Supplementary figures

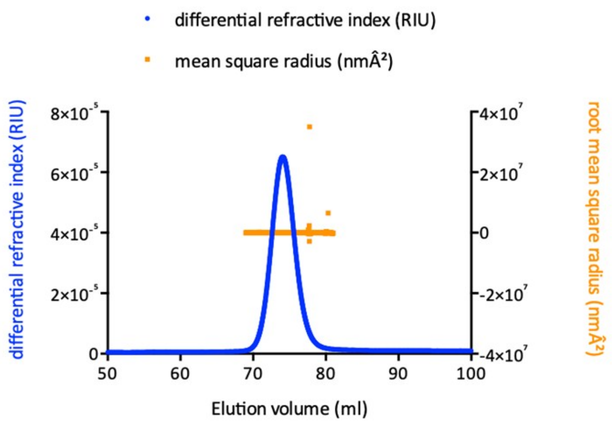

Figure S1 SEC-MALS chromatograph showing elution of the protein from gel filtration column. Changes in the refractive index (blue) show protein-mediated scattering of light. The root-mean-square radius (orange) allows estimation of the molecular size, Table S1.

```
3K28   ----MRKFDKSIAAFEEAQDLMPGGVNSPVRAFKSVGMNPLFMERGKGSKVYDIDGNEYI 56
ForI   -----MITEKSAALYARAVEVMPGGNSRTA---VYSSPYPVYVRSGSGARVTDVDGVERI 52
GSAAT  VTSSPFKTIKSDEIFAAAQKLMPGGVSSPVRAFKSVGGQPIVFDRVKDAYAWDVDGNRYI 60
MtIlvE ------------------------------------------------------------ 0
NeoB   -------MTKNSSLLAEFP-----------TCPRDEKDRPRVFTAASGAWLTDESGFRWI 42

3K28   DYVLSWGPLIHGHANDRVVEALKAVAE--RGTSFGAPTEIENKLAKLVIERVPSIEIVRM 114
ForI   DFLNNSTTLIHGHAHPEMVEAIAAAVG--HGTSFGMPTPVEVEYAEALSARNETFEHVRF 110
GSAAT  DYVGTWGPAICGHAHPEVIEALKVAME--KGTSFGAPCALENVLAEMVNDAVPSIEMVRF 118
MtIlvE -----------------------------------------------MTSGSLQFTVLRA 13
NeoB   DFDNARGSILLGHGDPVVAEAVARAATGADGTATGWSRRVDAVLERL--HALCGGEVVGL 100

3K28   VNSGTEATMSALRLARGYTGRNKILKFIGCYHGHGDSLLIKAGSGVATLGLPDSPGVPEG 174
ForI   TSSGTEAVMMAVQAARAYTERPKIAKIAGAYHGAYDAVAVNND-GSGNLISHAVTGNPEG 169
GSAAT  VNSGTEACMAVLRLMRAYTGRDKIIKFEGCYHGHADMFLVKAGSGVATLGLPSSPGVPKK 178
MtIlvE VNPATDAQRESMLREPG----------FGKYHT-DHMVSIDYAEGRGWHNARVIPYGPIE 62
NeoB   FRSGTAAVRAAVLAVREATGRPLLL--SAGYHGYDPMWYPSEA--------------PLE 144

3K28   VAKNTITVAYND--LESVKYAFEQFGDDIACVI-------VEPVAGNM-GVVPPQPGFLE 224
ForI   VVANTVVIPFND--PEGSLEVLRRHADDLACVL-------IDPVPWRI-GLLPASKEWLD 219
GSAAT  TTANTLTTPYND--LEAVKALFAENPGEIAGVI-------LEPIVGNS-GFIVPDAGFLE 228
MtIlvE LDPSAIVLHYAQEVFEGLKA-YRWADGSIVSFRADANAARLRSSARRLAIPELPDAVFIE 121
NeoB   PNADGVVDFFFD--LGLLRE-LLRAPERVAAVV-------VSPD------HMHLSPGWYR 188

3K28   GLREVTEQNGALL---------------IFDEVMTGFRVAYNCGQGYYGVTPDLTCLGKV 269
ForI   ALREFCDASGAVL---------------ISDEV-GSYRVGYHGAMQLLGAEADITVMGKV 263
GSAAT  GLREITLEHDALL---------------VFDEVMTGFRIAYGGVQEKFGVTPDLTTLGKI 273
MtIlvE SLRQLIAVDKAWVPGAGGEEALYLRPFIFATEPGLGVR--PATQYRYLLIASP---AGAY 176
NeoB   ELRRLCSAAGVVL---------------VADEVKVGLRYAPGLSTA-ELLAPDVWVVAKG 232

3K28   IGGGL-PVGAYGGKAEIMRQVAPSG---PIYQAGTLSGNPLAM----AAGYETLVQLT-P 320
ForI   IAAGM-PIGAVAGRRRFMSVFDPSKGKPALPHSGSYNANPVSM----SSGIASLRLLT-P 317
GSAAT  IGGGL-PVGAYGGKREIMQLVAPAG---PMYQAGTLSGNPLAM----TAGIKTLELLRQP 325
MtIlvE FKGGIAPVSVW-VSTEYVRAC-P-GGTGAAKFGGNYAASLLAQAEAAENGCDQVVWLD-A 232
NeoB   MANGH-AVSAVGGSRRLLKPLKEVSFT-SFF-------EPTIL----AAADAALARVATG 279

3K28   ESYVEFERKAEMLEAGLRKAAEKHGIPHHINRAGSMIGIFFTD-EP--------VI--NY 369
ForI   QAHERIGQLGEQARGSMRTALGEAGLDWEVNGLGSLFRVVANS-AP--------A---GY 365
GSAAT  GTYEYLDQITKRLSDGLLAIAQETGHAACGGQVSGMFGFFFTE-GP--------VH--NY 374
MtIlvE VERRYIEE------------------------MGGMNIFFVLGSGGSARLVTPELSGSLL 268
NeoB   EPQRAVREAGDRFLRHARKALDDASLPVEIAGDGTFFQFVPAT-EE--------LEEALY 330

3K28   DA-AKSSNLQFFAAYYREMVEQGVFLPPS-----------QFEGLFLST-------VHSD 410
ForI   DS-AA----AAMKALYWKLLENGIHIGDS-----------GLGCISTP---------MGE 400
GSAAT  ED-AKKSDLQKFSRFHRGMLEQGIYLAPS-----------QFEAGFTSL-------AHTE 415
MtIlvE PGITRDSLLQLAIDAGFAVEERRIDIDEWQKKAAAGEITEVFACGTAAVITPVARVRHGA 328
NeoB   GA-ANAEGLLFYAG-----DNQGV------------------SAAFDEA-------VLGE 359

3K28   ADIE-----------------ATIAAAEIAMSKL-----KA----------------- 429
ForI   EEI---------------------AEYAVAFAKSLGQV-LAEGRA------------- 423
GSAAT  EDID-----------------ATLAAARTVMSAL------------------------ 432
MtIlvE SEFRI----------ADGQP------GEVTMALRDTLTGIQRGTFADTHGWMARLG-- 368
NeoB   AERRFARVCERLAPYAGGEPVGDAARYRVAWNVMDGLR-QAPRDREETTGLLARLLDD 416
```

Figure S2 Sequence based alignment of ForI with 3K28, GSAAT, *Mt*Ilve and NeoB. ForI shows 30 %, 10 % and 24 % sequence identity with GSAAT, NeoB and *Mt*Ilve, respectively. 3K28 shows 50 % sequence similarity with ForI.

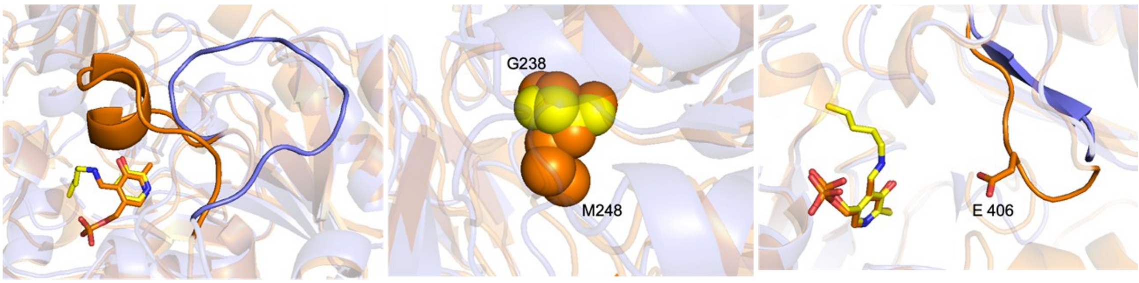

a b c

Figure S3 Structural analysis of active site residues of ForI (slate) and GSAAT (orange). (a) There is a α-helix that closes up the active site in GSAAT. While in ForI, the loop flips away opening the active site. PLP in ForI is colored in yellow and PMP in GSAAT colored in orange. (b) Met 248 in active site of GSAAT takes more space than Gly 238 in ForI. Met 248 from GSAAT in orange, Gly 238 in yellow. (c) The loop containing functionally important residue Glu 406 is close to the co-factor in GSAAT but kinks away from the active site in ForI.

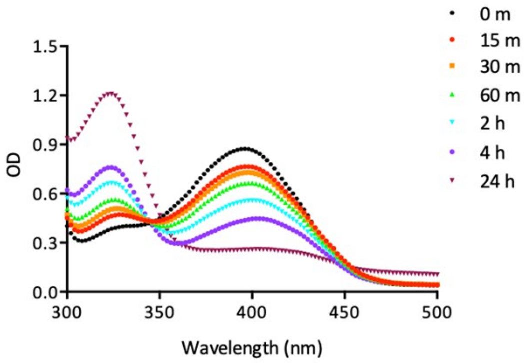

Figure S4 UV spectrum of ForI upon incubation with L-Glu. The decrease in intensity at the wavelength of 400 nm corresponding to the consumption of PLP and increase at the wavelength of 330 nm corresponding to the accumulation of PMP.

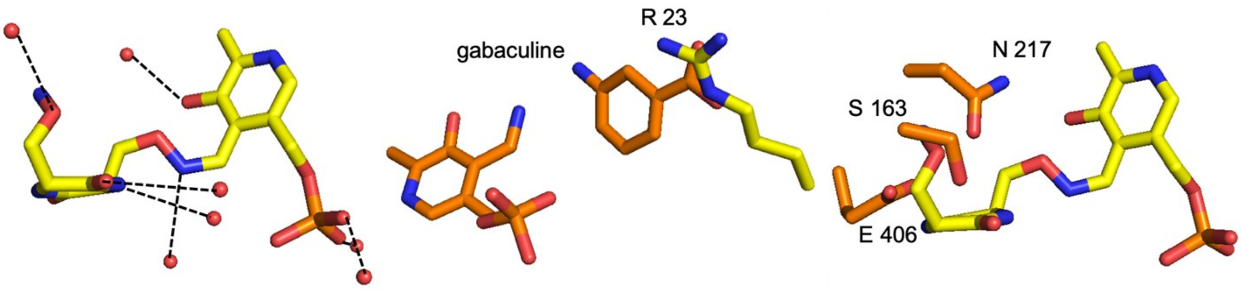

a b c

Figure S5 Active site structural analysis of ForI (a) L-configured diketopiperazine adduct hydrogen bonds with water molecules. (b) When GSAAT (PDB: 3GSB) and ForI are superimposed, Arg 23 of ForI would clash with gabaculing. (c) Ser 163, Asn 217, Glu 406 of GSAAT would block diketopiperazine binding at its active site. Residues from GSAAT are shown in orange and those from ForI are shown in yellow, with oxygen atoms in red and nitrogen atoms in blue.

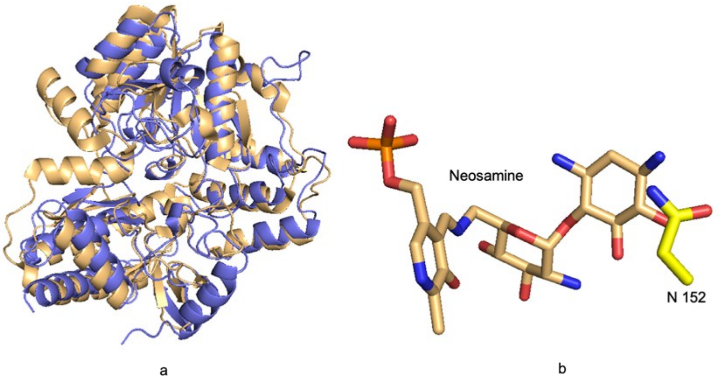

Figure S6 Structure analysis of NeoB (PDB: 6CBL) (pale orange) compared with ForI-LCS complex (slate). (a) Structure alignment of NeoB and ForI-LCS complex shows a high structural similarity with r.m.s.d. = 1.94 Å over 337 residues. (b) The six-membered carbohydrate ring of neosamine attached to the cofactor, sits in the catalytic pocket of ForI, while the other ring clashes with Asn 152 of ForI. Residues from NeoB are shown in pale orange and those from ForI are shown in yellow, with oxygen atoms in red and nitrogen atoms in blue.

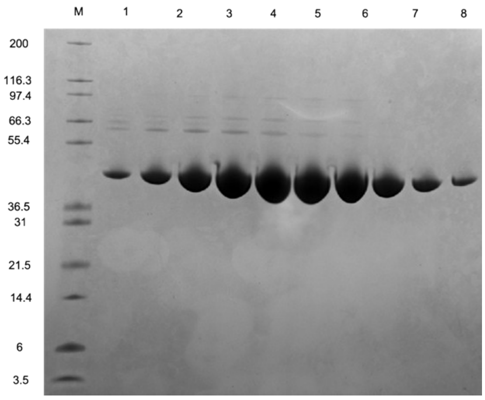

Figure S7 SDS-PAGE analysis of ForI. Coomassie blue stained SDS-PAGE of fractions from gel filtration. Lane 1-8 are elution fractions.

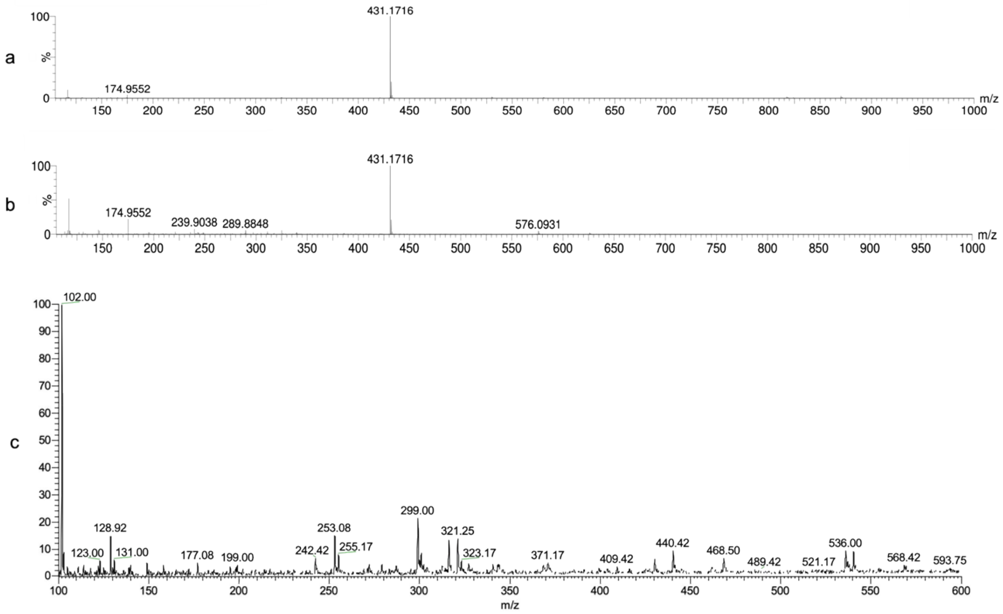

Figure S8 Mass spectra showing the presence of an ion (m/z = 431) corresponding to the anion of the diketopiperazine observed by X-ray crystallography. (a) and (b) shows the reaction of ForI (with 50 µM external PLP added) incubated with LCS and DCS, respectively. The details of the reaction are in materials. (c) In a solution of L-cycloserine (102 Da), no peak corresponding to diketopiperazine (204 Da) was observed.

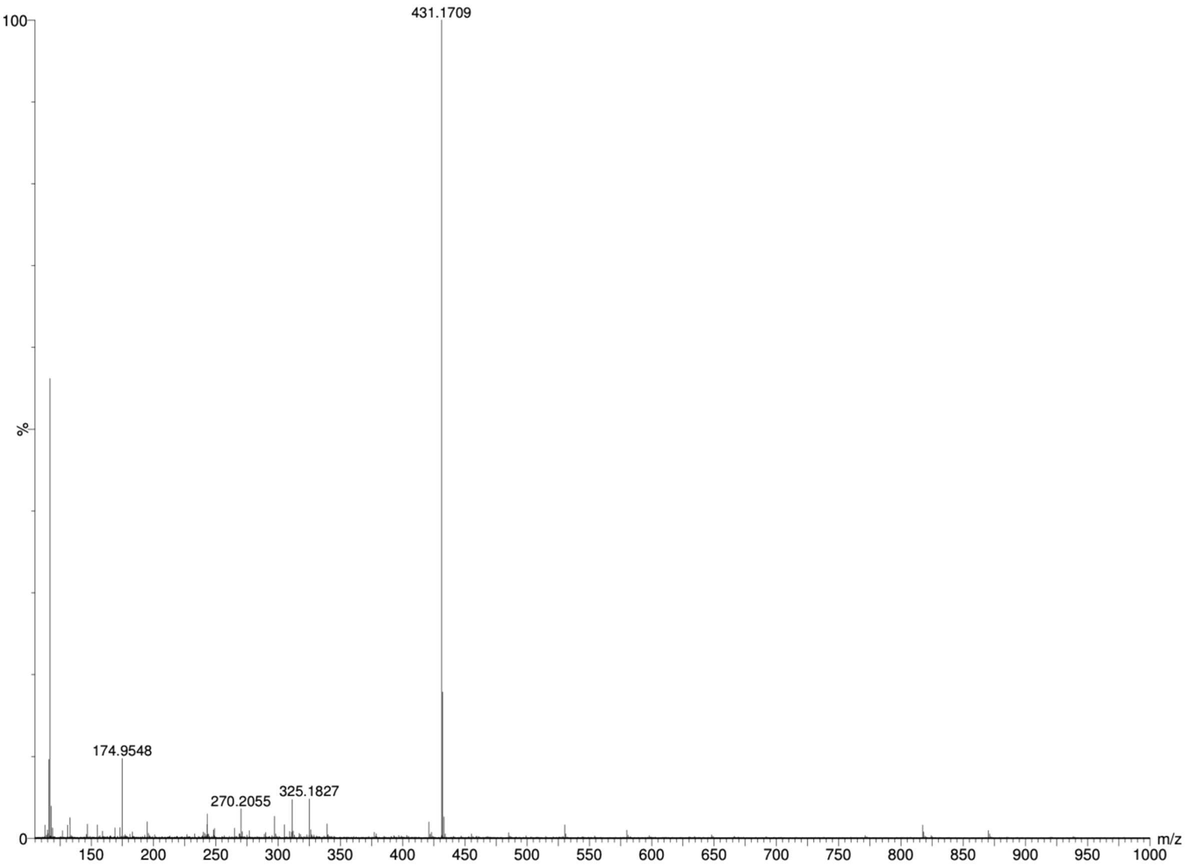

Figure S9 In a repeat of the experiment in Figure S8, the reaction was carried out in D_2O.The resulting mass spectrum shows the presence of an ion (m/z = 431) corresponding to the anion of the diketopiperazine but no incorporation of deuterium was observed, although the experiment was set up in D_2O.

Supplementary tables

Table S1 Experimentally derived mass for ForI in each SEC-MALS fraction. A ForI monomer has calculated molecular mass of 44kDa.

Fraction	Elution volume (ml)	Mw (kDa)	Uncertainty
1	70-71	353	± 5 %
2	71-72	113	± 17 %
3	72-73	87	± 7 %
4	73-74	83	± 3 %
5	74-75	81	± 3 %
6	75-76	80	± 4 %
7	76-77	90	± 13 %
8	77-78	200	± 60 %

Table S2 Data collection and refinement statistics.

	ForI-PLP	ForI-PMP	ForI-LCS	ForI-DCS
Beamline	i24 (DLS)	i04 (DLS)	i24 (DLS)	i24 (DLS)
Wavelength (Å)	0.9686	0.9795	0.9686	0.9686
Space group	$P4_32_12$	$P2_12_12_1$	$P2_12_12_1$	$P2_12_12_1$
Cell dimensions				
a, b, c (Å)	92.3 92.3 118.8	58.3 125.8 142.8	57.9 126.8 141.2	58.1 127.1 141.2
α, β, γ (°)	90 90 90	90 90 90	90 90 90	90 90 90
Resolution range	43.02-1.18 (1.21 - 1.18)	94.39 - 1.56 (1.60 - 1.56)	61.67 - 1.48 (1.52 - 1.48)	61.73 - 1.47 (1.51 - 1.47)
R_{meas}	0.070 (1.89)	0.182 (2.702)	0.160 (2.709)	0.173 (2.453)
$CC_{1/2}$	1.0 (0.5)	1.0 (0.5)	1.0 (0.7)	1.0 (0.7)
I/ σ (I)	22.3 (1.5)	8.0 (1.1)	16.8 (1.3)	13.9 (1.2)
Completeness (%)	93.1 (61.7)	98.8 (93.3)	98.0 (96.3)	97.9 (96.1)
Multiplicity	23.6 (16.5)	9.7 (9.7)	33.1 (29.6)	33.1 (28.7)
**Refinement**				
No. reflections	148310 (4974)	140719 (6922)	161265 (8227)	165228(8486)
R_{work}	0.112 (0.256)	0.167 (0.286)	0.142 (0.318)	0.145 (0.306)
R_{free}	0.129 (0.275)	0.192 (0.297)	0.173 (0.334)	0.169 (0.312)
Number of atoms				
Total	4051	7448	7473	7428
Protein	3385	6500	6502	6537
Ligands / ions	5	41	59	59
waters	661	907	912	832
B-factor				
Average	19.28	21.95	23.38	23.82
Protein only	15.1	18.4	20.2	20.6
Ligands/Ion/water	22.4	25.1	26.2	26.7
R.m.s deviations				
Bond lengths (Å)	0.012	0.013	0.005	0.005
Bond angles (°)	1.59	1.76	1.34	1.32

Values in parentheses are for the highest-resolution shell.
Structure and data have been deposited with RCSB.

Method and materials

General methods

Unless specified, all chemicals were purchased from Sigma-Aldrich or Fisher Scientific. Oligonucleotides were purchased from ThermoFisher Scientific. Restriction enzymes were purchased from New England Biolab. dNTP and DNA polymerase were purchased as part of the EMD Millipore Novagen KOD Hot Start DNA Polymerase kit. DNA sequencing was performed by GATC.

Cloning and expression

Streptomyces kaniharaensis Shomura and Niida (ATCC® 21070™) was purchased from ATCC (Middlesex, UK). The freeze-dried cell sample was directly dissolved in autoclaved Tryptic Soy Broth media containing 25% glycerol in biological safety cabinet. 10 μl of the cell culture was plated on the TSB agar and incubate at 26 °C for 15 days. Four 0.5-inch diameter mycelia appeared on the plate, and one of the mycelia was picked for inoculating 50 mL TSB liquid media. The cell culture was incubated at 26 °C for 5 days before harvesting by centrifugation at 3500 rpm at 4 °C. The cell pellets were washed by deionized water twice before store in -80 °C overnight. The genomic DNA extraction was performed using PureLink™ Genomic DNA Mini Kit (ThermoFisher, UK) and the manufacture protocol for Gram-positive bacteria. To increase the purity, the yield genomic DNA was purified again using the same procedure. This yielded 17 μg genomic DNA (260nm/280nm = 1.9, 260nm/230nm = 2.0) for sequencing. Preparation of PacBio DNA library from the genomic DNA sample, data generation on RSII SMRT cell, and genome assembly using HGAP were performed at University of Liverpool Centre for Genomic Research. The final assembly of the genome of *S. kaniharaensis* contained 19 contigs. The genome was annotated through the PATRIC software pipeline (https://www.patricbrc.org/).[1] The final assembly of the genome of *S. kaniharaensis* contained 19 contigs. The genome sequence has been deposited with accession number is: SAMN12859417. We tentatively identified the gene cluster encoding by searching for the homologs of the known formycin synthesis protein ForH (PDB id: 6NKO) using the internal PATRIC BlastP tool and then analyzing the surrounding CDSs for homologs of previously identified *forA-X* genes.[2] The ForI protein was predicted based on the membership to the PLP-dependent aminotransferase family (fig|212423.3.peg.657). The corresponding codon optimised synthetic gene ForI was purchased from Integrated DNA Technologies, Europe and then cloned into a pEHISTEV vector[3] resulting in a plasmid with the coding DNA sequence below.

The codon optimised synthetic gene ForI[2] was purchased from Integrated DNA Technologies, Europe and then cloned into a pEHISTEV vector[3] resulting in a plasmid with the coding DNA sequence below.

```
1       ATGTCGTACT ACCATCACCA TCACCATCAC GATTACGACA TCCCAACGAC CGAAAACCTG
61      TATTTTCAGG GCGCCATGAT AACAGAGAAG TCTGCCGCCT TATACGCCCG GGCCGTAGAG
121     GTCATGCCTG GCGGTAATTC GAGAACTGCC GTATATTCTT CTCCATATCC GGTATATGTA
181     CGTAGCGGCT CTGGAGCGAG AGTTACGGAC GTTGACGGAG TTGAAAGAAT CGACTTTTTG
241     AACAATTCGA CCACTTTGAT TCACGGGCAC GCCCACCCGG AAATGGTCGA GGCAATCGCG
301     GCCGCTGTTG GACATGGCAC GTCTTTTGGA ATGCCTACAC CGGTTGAAGT TGAATACGCT
361     GAGGCTTTAA GCGCGCGCAA CGAAACATTC GAACATGTGA GATTTACGTC GAGCGGAACA
421     GAAGCTGTAA TGATGGCTGT GCAAGCTGCG CGGGCATATA CGGAGCGGCC AAAAATTGCT
481     AAGATTGCTG GCGCCTATCA TGGCGCGTAT GATGCTGTCG CCGTCAACAA CGATGGTTCC
541     GGGAACCTTA TCAGCCATGC AGTGACCGGG AATCCCGAAG GGGTCGTAGC TAACACTGTC
601     GTAATTCCGT TTAACGACCC AGAAGGTAGC TTGGAAGTAT TACGTCGTCA CGCCGATGAT
661     CTGGCGTGTG TATTAATTGA CCCCGTACCA TGGCGCATCG GCCTTTTGCC TGCATCGAAG
721     GAGTGGTTAG ACGCTCTTCG TGAGTTCTGT GATGCCTCTG GAGCCGTGTT GATTAGCGAT
781     GAGGTAGGTT CATATCGGGT AGGCTACCAT GGAGCCATGC AACTTCTGGG CGCCGAGGCG
841     GATATTACCG TGATGGGAAA AGTAATAGCC GCAGGTATGC CTATAGGAGC TGTAGCTGGC
901     AGACGGCGTT TCATGTCCGT ATTCGATCCG AGCAAAGGCA AGCCTGCCTT GCCACACAGT
961     GGATCGTACA ATGCAAATCC TGTTTCCATG TCTTCAGGAA TCGCTAGTCT GCGGCTTTTG
1021    ACTCCTCAAG CGCATGAGAG AATCGGTCAA TTGGGTGAGC AGGCTAGAGG TAGTATGCGC
1081    ACAGCTTTGG GTGAGGCGGG ATTGGACTGG GAAGTGAATG GTTTGGGTAG CTTGTTTCGC
1141    GTCGTAGCTA ATTCGGCACC AGCCGGATAC GACAGTGCAG CAGCCGCTAT GAAGGCTCTT
1201    TATTGGAAGT TACTGGAGAA CGGAATCCAC ATAGGTGATT CGGGGCTTGG GTGCATTTCC
1261    ACGCCGATGG GAGAAGAGGA AATAGCGGAG TACGCCGTAG CGTTTGCGAA GAGTTTGGGC
1321    CAGGTATTGG CGGAAGGCCG TGCCTAG
```

(highlighted in cyan are the His tag and TEV cleavage site).

The plasmid has been deposited with ADDGENE. This construct has an N terminal His_6 tag followed by a tobacco etch virus cleavage site before the start of the protein. The resulting protein was expressed in *Escherichia coli* BL21(DE3) cells grown in the autoinduction media described by Studier[4] for 48 h at 20 °C.

Protein purification

E. coli cells overexpressing ForI were resuspended in lysis buffer [500 mM NaCl, 20 mM Tris-HCl (pH 8.0), 20 mM imidazole (pH 8.0), and 3 mM 2-mercaptoethanol] and EDTA-free protease inhibitor tablets (Roche) and DNase at 0.4 mg/g of wet cell pellet. The resuspension was lysed by being passed through a cell disruptor at 30K psi (Constant Systems). The lysate was cleared by centrifugation (17,000 rpm, 4 °C, 20 min) and then loaded onto a Ni Sepharose 6 Fast Flow column (GE Healthcare) equilibrated with lysis buffer. The protein was eluted with elution buffer [500 mM NaCl, 20 mM Tris-HCl (pH 8.0), 250 mM imidazole (pH 8.0), and 3 mM 2-mercaptoethanol] and passed over a desalting column (16/10 Desalting, GE Healthcare) exchanging into desalting buffer [100 mM NaCl, 20 mM Tris-HCl (pH 8.0), 3 mM 2-mercaptoethanol]. TEV protease was added at a mass ratio of 1:10, and

the protein was digested for 3 h at 20 °C before being loaded onto a second nickel column pre-equilibrated with desalting buffer. The eluted protein was applied directly to an anion-exchange column (HisTrap Q Sepharose FF, GF Healthcare) where it was eluted with a 0.1 to 1 M NaCl gradient. The peak fraction was then concentrated to 7.5 mL (Vivaspin concentrators, 30 kDa molecular weight cutoff) and applied to a Superdex 200 gel filtration column (GE Healthcare) equilibrated with gel filtration buffer [150 mM NaCl, 20 mM HEPES (pH 7.4), and 1 mM TCEP]. The integrity, identity and purity of ForI was confirmed by SDS gel electrophoresis (Figure S7) and MS. 50 µM external PLP is added in the protein before it was fast frozen by liquid nitrogen.

UV – vis absorbance spectroscopy of ForI.

All UV absorbance spectra were recorded on a SpectraMax 2e microplate reader (Molecular Devices) and analysed using Graphpad Prism 6. An additional 1 µM PLP was added to enzyme to aid protein stability. For UV - vis absorbance assays, the concentration of recombinant protein was 50 µM. The plate reader was blanked with 100 mM HEPES (pH 7.5) and spectra were collected from 300 nm to 500 nm. The screening experiments used a total sample volume of 100 µl. The instrument was set to record spectra at a wavelength step of 2 nm. Changes in the spectrum were monitored after addition of 0.5 mM L-glutamate or 5 mM cycloserine (D/L).

Oligomeric status of ForI determination

ForI was analysed by size-exclusion chromatography multi-angle light scattering (SEC-MALS)[5] for determination of molecular mass. Purified protein was loaded onto a GE Health Superdex 200 column, equilibrated in Gel filtration buffer (150 mM NaCl, 10 mM HEPES, 1 mM TCEP), attached to the Wyatt Dawn Heleos II Multi-Angle Light Scattering detector and Wyatt Optilab T-rex Refractive Index detector. The protein elution peak was characterised by the differential refractive index (dRI).

Crystallography

The enzyme was screened for suitable crystallisation conditions. 1 mM PLP was added into protein to ensure a complete loading prior to crystallisation. Crystals were obtained in 200 mM Li_2SO_4, 100 mM Tris-HCl pH 8.0 and 30 % (w/v) PEG 4000 with 35 mg/ml protein at 4 °C. The original hit was optimised in hanging drop plates (EasyXtal 15-well DG-Tool X-Seal) using 1 µl of the protein solution (35 mg/ml) and 1-2 µl of well solution in the hanging drop. The PMP form of ForI was produced by soaking the crystals in the mother liquor plus 10 mM L-glutamic acid for 5 min at 4 °C or incubating 10 mM L-glutamic acid with enzyme for 16 hours at 4 °C prior to crystallisation. For the preparation

of L- and D-cycloserine crystals, 10 mM of the corresponding cycloserine was incubated with the protein at 4 °C for 16 hours before setting up crystallisation plates. Crystals were harvested at 3 days later. The crystallisation conditions were identical to those optimised for the ForI-PLP but the space group was different.

The crystals were mounted in a cryo-loop (Molecular Dimensions) and cryo-protected in solutions containing mother liquor brought to 20 % (v/v) glycerol. The crystals were then frozen by plunging them into liquid nitrogen and sent in a cryogenic Dewar to Diamond Light Source for data collection. The data sets were collected at two different beam lines (I04 and I24) and all data processing used the Diamond online automated software XIA2[6] DIALS[7]. The resolution limit of data is determined where $CC_{1/2} \geq 0.5$ [8] and intensity fall off. Structures were determined using molecular replacement in PHASER[9] (the native ForI used the GSAAT as search model, subsequent and refined in REFMAC5[10] with anisotropic B-factors as implemented in CCP4.[11] Ligands were introduced into density during refinement when the Fo-Fc map was judged unambiguous in COOT.[12] The maps that we introduced the ligands into are the same omit maps that we showed in the paper. Thus the phases for these Fo-Fc maps were derived from models that never had the ligand present. The final refined coordinates of the ligand were then shown in this Fo-Fc map. This method of calculating the omit maps is unbiased. The geometry of the diketopiperazine was restrained guided by parameters from the PRODRG server.[13] Data and structures have been deposited with the RCSB.

Mass spectrometric detection of diketopiperazines

ForI, dissolved in 20 mM HEPES buffer, pH 7.4, containing 150 mM NaCl was passed through a desalting column (GE Healthcare) to exchange the protein into 100 mM NH_4OAc, pH 8.0. 2 mM of either L- or D-cycloserine and 100 μM ForI dissolved in 100 mM NH_4OAc, pH 8.0, (50 μL total volume) were then incubated overnight at 4 °C before the addition of MeOH (50 μL) to precipitate the enzyme. Centrifugation then gave a supernatant that was analysed by mass spectrometry. Control samples lacking either the enzyme or cycloserine were prepared in a similar manner. Liquid chromatography mass spectrometry (LC-MS) was performed on a Waters Acquity UPLC coupled to a Waters Synapt G2-Si QTOF mass spectrometer. The column used was a Waters Acquity CSH C18, 130 Å, 1.7 μm (2.1 mm x 30 mm) and held at 40 °C throughout the run. Mobile phase A was H_2O (0.1% CHOOH) and B was ACN (0.1% CHOOH). The flow rate was 0.3 mL/min and the gradient employed began at 98% A with a final of composition of 2% A over 10 minutes. The mass spectrometer was operated in electrospray ionisation positive or negative mode. Negative ion mass spectrometric analysis of both ForI/cycloserine reaction mixtures clearly showed ions with m/z =

431 corresponding to the anion of the corresponding diketopiperazine distereoisomer (Figure S8). This peak was absent in the corresponding mass spectra of the control samples of protein nor was any peak corresponding to diketopiperazine observed.

For deuterium experiment, ForI was exchanged into 100 mM NH_4OAc, pH 8.0 in D_2O. 2 mM L-cycloserine (in D_2O) and 100 μM ForI dissolved in 100 mM NH_4OAc, pH 8.0, (50 μL total volume) were then incubated overnight at 4 °C before the addition of MeOH (50 μL) to precipitate the enzyme. Centrifugation then gave a supernatant that was analysed by mass spectrometry.

References

1 Wattam AR, Davis JJ, Assaf R, Boisvert S, Brettin T, Bun C, Conrad N, Dietrich EM, Disz T, Gabbard JL, Gerdes S, Henry CS, Kenyon RW, Machi D, Mao C, Nordberg EK, Yoo H, Stevens RL. *Nucleic Acids Res*. 2017, **45**, D535–42.
2 Wang S-A, Ko Y, Zeng J, Geng Y, Ren D, Ogasawara Y, Irani S, Zhang Y, Liu H. *J Am Chem Soc*. 2019, **141**, 6127–31.
3 Liu H, Naismith JH. *Protein Expr Purif*. 2009, **63**, 102–11.
4 Studier FW. *Protein Expr Purif*. 2005, **41**, 207–34.
5 Tarazona MP, Saiz E. *J Biochem Biophys Methods*. 2003, **56**, 95–116.
6 Winter G, IUCr. *J Appl Crystallogr*. 2010, **43**, 186–90.
7 Winter G, Waterman DG, Parkhurst JM, Brewster AS, Gildea RJ, Gerstel M, Fuentes-Montero L, Vollmar M, Michels-Clark T, Young ID, Sauter NK, Evans G. *Acta Crystallogr Sect D Struct Biol*. 2018, **74**, 85–97.
8 Karplus PA, Diederichs K. *Curr Opin Struct Biol*. 2015, **34**, 60–8.
9 McCoy AJ, Grosse-Kunstleve RW, Adams PD, Winn MD, Storoni LC, Read RJ, IUCr. *J Appl Crystallogr*. 2007, **40**, 658–74.
10 Murshudov GN, Vagin AA, Dodson EJ, IUCr. *Acta Crystallogr Sect D Biol Crystallogr*. 1997, **53**, 240–55.
11 Winn MD, Ballard CC, Cowtan KD, Dodson EJ, Emsley P, Evans PR, Keegan RM, Krissinel EB, Leslie AGW, McCoy A, McNicholas SJ, Murshudov GN, Pannu NS, Potterton EA, Powell HR, Read RJ, Vagin A, Wilson KS, IUCr. *Acta Crystallogr Sect D Biol Crystallogr*. 2011, **67**, 235–42.
12 Emsley P, Cowtan K, IUCr. *Acta Crystallogr Sect D Biol Crystallogr*. 2004, **60**, 2126–32.
13 Schüttelkopf AW, van Aalten DMF, IUCr. *Acta Crystallogr Sect D Biol Crystallogr*. 2004, **60**, 1355–63.
